# Mutagenic and Genotoxic Effect of Hydroxyurea

**Published:** 2011-12

**Authors:** Jean L. Santos, Priscila L. Bosquesi, Adélia E. Almeida, Chung Man Chin, Eliana A. Varanda

**Affiliations:** 1 *Lapdesf - Laboratório de Pesquisa e Desenvolvimento de Fármacos. Departamento de Fármacos e Medicamentos - Faculdade de Ciências Farmacêuticas - Universidade Estadual Paulista “Júlio de Mesquita Filho” (UNESP) Rodovia Araraquara-Jaú Km.01 s/n, 14801-902, Araraquara, São Paulo, Brasil;*; 2 *Departamento de Ciências Biológicas - Faculdade de Ciências Farmacêuticas - Universidade Estadual Paulista “Júlio de Mesquita Filho” (UNESP) Rodovia Araraquara-Jaú Km.01 s/n, 14801-902, Araraquara, São Paulo, Brasil*

**Keywords:** hydroxyurea, mutagenicity, genotoxicity, micronucleus test

## Abstract

The hydroxyurea, a cytotoxic drug, is the mainly available therapeutical strategy for the treatment of sickle cell disease. This study aimed to evaluate the mutagenic and genotoxic potential of the hydroxyurea through the *Salmonella*/Microsome assay and micronucleus test in peripheral blood of mice. The doses were evaluated at 29.25-468 μmol/plate in *Salmonella*/Microsome assay in presence and absence of metabolic activation the drug. In the micronucleus test the doses were evaluated at 12.5; 25; 50; 75 and 100 mg/kg. The results show that hydroxyurea present mutagenic activity in TA98 and TA100 in doses above 117 μmol/plate and 234 μmol/plate respectively. The drug induced a significant increase in the frequency of micronuclei in reticulocytes of mice at concentrations of 50, 75 and 100 mg/kg, compared to negative control (water). These results demonstrated the mutagenic and genotoxic potential of hydroxyurea.

## INTRODUCTION

Hydroxyurea (HU) is an antineoplastic and a chemotherapeutic agent widely used for the treatment of myeloproliferative diseases, including myeloid leukaemia, polycythemia vera and essential thrombocytopenia. This drug has been utilized for at least two decades in the treatment of sickle cell disease (SCD). HU may be given to children and adults with SCD for an extended period of time or for repeated cycles of therapy and its beneficial effect in SCD has been associated with its capacity to induce fetal hemoglobin synthesis (1).

Despite of this beneficial effect, it knows that the treatment with HU is associated with known side effects such as cytotoxicity and myelosuppression. In the literature, the HU ability to cause cancer is controversial and the long-term efficacy and safety of HU in treating patients with SCA remain incompletely defined (2). Some studies have shown that hydroxyurea is genotoxic while other studies suggest that hydroxyurea has low mutagenicity *in vivo* (3, 4).

The fact is that HU inhibited the enzyme ribonucleotide reductase interrupting the normal mechanism of ribonucleotides and deoxyribonucleotides reduction and in the last step limiting DNA biosynthesis (3, 4). Some reports related that HU acts as a competitive inhibitor of catalase-mediated hydrogen peroxide decomposition and this effect could be related to *in vivo* toxicity (5). In eukaryotic cell, a study of HU, in mammalian (V79) cells, reported microsomal activation-dependent mutagenicity and found that the addition of catalase inhibited microsome mediated mutagenicity, indicating that hydrogen peroxide was involved in the formation of mutagenic DNA lesion (6).

The assessment of genotoxicity through standardized tests recommended by regulatory agencies is crucial to enable a safer and more adequate therapy. Based on clinical evidence, antineoplasic agents induce tumours when used at therapeutic doses, mainly acute non-lymphocytic leukaemia (7). So, the evaluation of genotoxicity is essential. The aim of this present study was to evaluate the mutagenic and genotoxic effect of HU using *Salmonella*/Microsome assay and micronucleus test.

## MATERIALS AND METHODS

### Chemicals

Hydroxyurea (HU, CAS No. 127-07-1), cyclophosphamide (CAS 50-18-0), acridine orange (CAS 65-61-2) dimethylsulfoxide (DMSO, CAS No. 67-68-5), nicotinamide adenine dinucleotide phosphate sodium salt (CAS No. 11-84-16-3), D-glucose-6-phosphate disodium salt (CAS No. 3671-99-6), magnesium chloride (CAS No. 7786-30-3), L-histidine monohydrate (CAS No. 7048-02-4), D-biotin (CAS No. 58-85-5), sodium azide (CAS No. 26628-22-8), 2-anthramine (CAS No. 613-13-8) and 2-aminofluorene (CAS No. 153-78-6) were purchased from Sigma Chemical Co. (St. Louis, MO, USA). Oxoid Nutrient Broth No. 2 (Oxoid, England) and Difco Bacto Agar (Difco, USA) were used as bacterial media. D-Glucose (CAS No. 154-17-6), magnesium sulfate (CAS No. 7487-88-9), citric acid monohydrate (CAS No. 5949-29-1), potassium phosphate dibasic anhydrous (CAS No. 7758-11-4), sodium ammonium phosphate (CAS No. 13011-54-6), sodium phosphate monobasic (CAS No. 7558-80-7), sodium phosphate dibasic (CAS No. 7558-79-4), sodium chloride (CAS No. 7647-14-5) were purchased from Merck (Whitehouse Station, NJ, USA).

### Animals

For the evaluation of *in vivo* mutagenicity of the drug hydroxyurea was used micronucleus test in peripheral blood cells of mice *Mus musculus* (Swiss albino) with about 30g of body weight, from the Central Animal Universidade Estadual Paulista - Unesp. The animals were kept in cages during the treatment period, with food and water *ad libitum*, light/dark cycle of 12 hours and temperature 23 ± 2°C. The research project for the development of this work was submitted to the Ethics Committee in Research of the Faculty of Pharmaceutical Sciences UNESP, Araraquara, São Paulo, Brazil, with all the methodology of animal experimentation approved: Protocol 36/2007.

### Metabolic Activation System (S9 Mixture)

The S9 fraction, prepared from livers of Sprague-Dawley rats treated with the polychlorinated biphenyl mixture Aroclor 1254, was purchased from Molecular Toxicology Inc. (Annapolis, MD, USA). The metabolic activation system consisted of 4% of S9 fraction, 1% of 0.4 M MgCl_2_, 1% of 1.65 M KCl, 0.5% of 1M D-glucose-6-phosphate disodium and 4% of 0.1 M b-nicotinamide adenine dinucleotide phosphate sodium in 0.1 M, 50% of 0.2 M phosphate buffer and 39.5% of sterile distilled water (8).

### Bacterial Strains

TA98 and TA100 strains of *Salmonella typhimurium* were kindly supplied by Dr Bruce N. Ames from The University of California, Berkeley, USA. For all assays, an inoculum (200 μL) of a thawed permanent culture was added to 20 mL of Oxoid Nutrient Broth No. 2 and incubated at 37°C with shaking until a concentration of approximately 1-2 × 10^9^ bacteria per milliliter was obtained.

### Mutagenicity Assay

The Salmonella mutagenicity assay was performed by pre-incubating the test compounds for 20-30 min with *Salmonella typhimurium* strains TA98 and TA100, with and without metabolic activation (S9 mixture). HU was tested at the following concentrations: 29.25; 58.5; 117; 234; 468 μmol per plate.

These doses were determined after the toxicity tests had been carried out. In all subsequent assays, the upper limit of the dose range tested was either the highest non-toxic dose or the lowest toxic dose determined in this preliminary assay. Toxicity was apparent either as a reduction in the number of his ± revertants, or as an alteration in the auxotrophic background (*i.e.*, background lawn).

The various concentrations of compounds to be tested were added to 500 μL of buffer pH7.4 and 100 μL of bacterial culture and then incubated at 37°C for 20-30 min. After this time 2 mL of top agar was added to the mixture and poured on to a plate containing minimum agar. The plates were incubated at 37°C for 48 h and the his ± revertant colonies were manually counted. The influence of metabolic activation was tested by adding 500 μL of S9 mixture (5%). All experiments were performed in triplicate.

The standard mutagens used as positive controls in experiments without S9 mix were sodium azide (1.25 μg/plate) for TA100 and 2-antramine (3 μg/plate) for TA98. 2-Anthramine (1.25 μg/plate) was used with TA100 and nitrophelylenediamine (3 μg/plate) with TA98 in the experiments with metabolic activation. DMSO served as the negative (solvent) control (100 μL/plate).

The statistical analysis was performed with the Salanal computer program, adopting the Bernstein model (9). The mutagenic index (MI)-the average number of revertants per plate divided by the average number of revertants per plate from the negative (solvent) control-was also calculated for each dose. A sample was considered positive when the MI was equal to or greater than 2 for at least one of the tested doses and if it had a reproducible dose-response curve (10).

### Evaluation of The Mutagenicity by Micronucleus Test in Peripheral Blood Cells of Mice

The doses were evaluated of drug hydroxyurea (12.5 25, 50, 75 and 100 mg/kg) via gavage administered to animals. For the treatment the animals were divided into groups of 10, with 5 males and 5 females. It was established a positive control group, in which the animals were intraperitoneally treated with cyclophosphamide (50 mg/kg) and a negative control group, where the animals received only water. The protocol adopted for this work was previously described (11), it which employ pre-stained laminas by Acridine Orange. After 30 hours, the animals were killed to collect the blood. We counted 1000 reticulocytes per animal and recorded the frequencies of micronucleated cells. After the cytological analysis of the laminas containing samples of peripheral blood of mice treated with the drug, were calculated frequency average cell micronuclei, and the standard deviations for each treatment groups. From these results was applied a test of analysis of variance (ANOVA). Where *P*<0.05, the average of treatments were compared using the test t, calculating the minimum significant difference for α=0.05.

## RESULTS

Table 1 shows the number of revertants/plate, the standard deviation and the mutagenic index (MI) after the treatments with the compounds, in the two different strains of *Salmonella typhimurium*, with or without metabolic activation.

**Table 1:** Mutagenic activity expressed as the mean and standard deviation of the number of revertants/plate in bacterial strains TA98 and TA100 exposed to HU, at various doses, with (+S9) or without (-S9) metabolic activation

Compounds	Concentration (μmol/plate)	Revertants/plate in *Salmonella typhimurium* strains			
		TA98		TA100	
		+S9	-S9	+S9	-S9

Hu	0	22.5 ± 1.0	26 ± 1.0	129.3 ± 8.1	143 ± 4.8
	29.25	37.3 ± 2.5 (1.6)	27.3 ± 2.5 (1.0)	178.33 ± 15.1* (1.4)	149 ± 8.9 (1.0)
	58.5	41.2 ± 2.3** (1.8)	33.7 ± 2.3** (1.3)	240 ± 3** (1.8)	159 ± 11 (1.1)
	117	50.2 ± 1.8** (2.2)	37.2 ± 1.8** (1.4)	220.7 ± 17.8** (1.7)	268.7 ± 13.5* (1.9)
	234	59.1 ± 2.7** (2.6)	41.1 ± 2.7** (1.5)	276 ± 27.7** (2.1)	345 ± 21.2** (2.4)
	468	43.3 ± 3.1** (1.9)	43.3 ± 3.1** (1.6)	315.3 ± 16.1** (2.4)	358.6 ± 5.5** (2.5)

0, negative control (DMSO–100 μL/plate)

* *P*<0.01 or

** *P*<0.05 (ANOVA).

The values in parenthesis = mutagenic index. Numbers represent averages of triplicates from the three different experiments ± the standard deviation. Positive control: sodium azide (1.25 μg/plate) for TA100 (-S9), 2-antramine (3 μg/plate) for TA98 (-S9) and 2-anthramine (1.25 μg/plate) for TA100 (+S9) and nitrophenylenodiamine (3 μg/plate) for TA98 (+S9).

HU exhibited mutagenicity in strains TA98 and TA100, in the last the mutagenicity was observed in presence and absence of metabolic activation. Using TA 98, the mutagenicity was observed only in presence of metabolic activation at 117 and 234 μmol/plate with mutagenic index of 2.2 and 2.6 respectively (Table 1). The sample is considered positive (mutagenic) when the mutagenic index is equal to or greater than 2 for at least one of the tested doses. In TA98 strain the concentration of 468 μmol/plate induced a reduction in number of revertants, consequence of the toxicity caused by HU. Using TA100, mutagenic index higher than 2 was observed at concentrations above 234 μmol/plate in presence and absence of metabolic activation. These results indicate that HU has a mutagenic potential at high concentrations above 234 μmol/plate using TA100 *Salmonella* strain.

**Figure 1:**
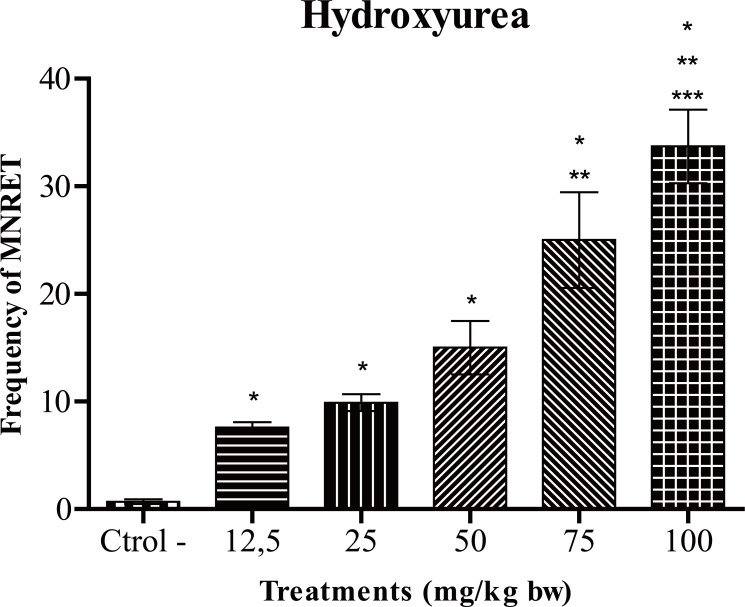
Average frequency of micronucleated reticulocytes (MNRET) and standard deviation of 1000 cells obtained from mice treated with water (negative control) and hydroxyurea. ^*^P<0.05 (compared to negative control), ^**^P<0.05 (compared to doses of 12.5 and 25 mg/kg), ^***^P<0.05 (compared to the doses 12.5, 25 and 50 mg/kg).

**Figure 2:**
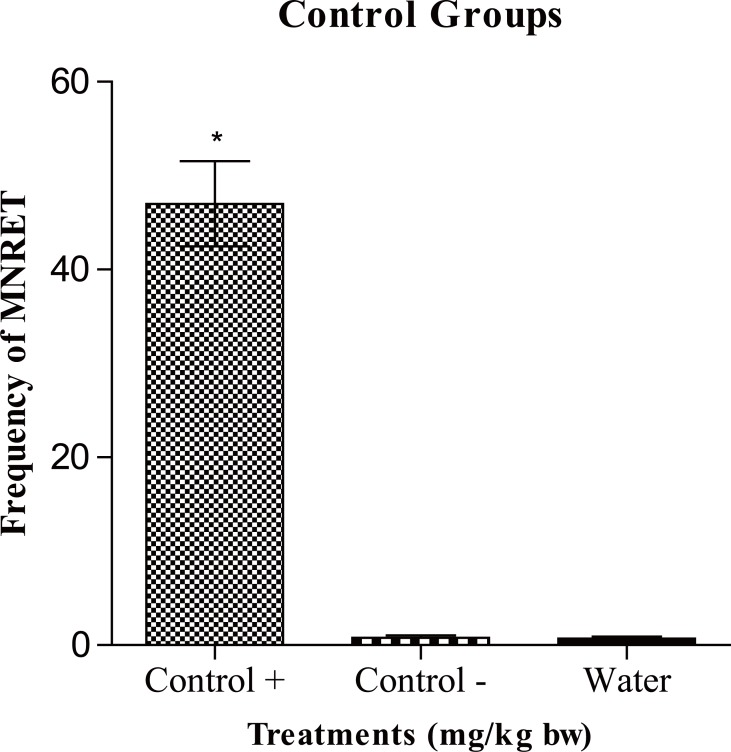
Average frequency of micronucleated reticulocytes (MNRET) and standard deviation of 1000 cells from mice treated with cyclophosphamide (positive control), CMC/Tween (negative control) and water (control white). ^*^P<0.05.

Figure 1 shows the frequency of micronucleated reticulocytes (MNRET) and standard deviation of 1000 cells obtained from mice treated with water (negative control) and hydroxyurea. The results show that the drug hydroxyurea, administered at different concentrations induced a significant increase in the frequency of micronuclei in reticulocytes of mice at all tested concentrations, compared to negative control (water). Cyclophosphamide used as positive control presented an average of 46 micronucleated reticulocytes (Figure 2). This result is not statically significant when compared to 100 mg/kg that presented an average of 34 micronucleated reticulocytes per slide.

Experiments were carried out at concentrations of 200 mg/kg and 400 mg/kg. The statistical analysis no show difference of the micronuclei frequency in a dose of 200 mg/kg when compared to 100 mg/kg. At a dose of 400 mg/kg was observed a decrease in the number of cells, possibly due to toxicity of hydroxyurea (results no showed).

## DISCUSSION

The mutagenicity and genotoxicity evaluation is essential to determinate the drug security for human use. Some assays as *Salmonella*/Microsome and micronucleus test are recommended by regulatory agencies. The *Salmonella*/Microsome is based upon the reversion of mutations in the histidine (his) operon in the *Salmonella typhimurium*. Strains with mutations in the his operon are histidine auxotrophs. In the presence of mutagenic compound revertants will restore the his^+^ phenotype and the bacteria will grow on minimal medium plates without histidine. The number of colonies produced is proportional to compound capacity to induce mutagenicity. The strain TA98 and TA100 respectively detect mutagens that cause displacement of frame reading and replacement of base pairs in DNA (8). Using *Salmonella*/Microsome assay our findings showed that HU present mutagenic potential although a previous study had showed the absence of mutagenicity of HU tested up 0.5 mg per plate in TA100, TA98 and TA1537 (12). Specifically, we demonstrated that mutagens that cause displacement of frame reading occur in presence of metabolic activation above 117 μmol/plate. This mutagenicity could be due to generation of some specie generating during HU metabolism. When analyzing the strain that detected replacement of base pairs in DNA (TA100), we observed that mutagenicity occur above 234 μmol/plate in presence and absence of metabolic activation.

The micronuclei test is a model to study genotoxicity used to detect and measure micronucleated reticulocytes. During normal erythropoiesis, red blood cells extrude their nucleus as they develop into functional reticulocytes. Occasionally, membrane bound DNA resulting from double-strand breaks or else lagging whole chromosomes remains in the cell after erythrocyte maturation and these inclusion bodies are known as micronuclei. During normal erythropoiesis, micronuclei are formed at low frequency, and MN-containing erythrocytes are removed quickly and efficiently from peripheral blood circulation. The presence of micronuclei in circulation indicates chromosomal damage (13).

Our results showed that HU present genotoxic potential in mammals’ cells at all concentrations tested. These results are consistent with previous study that had demonstrated that HU present genotoxicy using Cometa assay, although this assay has the disadvantage to not detect aneuploidy, chromosome rearrangement, DNA mis-repair or DNA adducts (13). Other study has related the HU genotoxicity to its capacity to generate indirectly hydrogen peroxide probably due to inhibition of catalase-mediated hydrogen peroxide decomposition (5, 6).

These results associated with reports in literature allow us to state that HU is a genotoxic agent and a presumable trans-species carcinogen.

## References

[1] Segal JB, Strouse JJ, Beach MC, Haywood C (2008). Hydroxyurea for the treatment of sickle cell disease. Evid. Rep. Technol. Assess.

[2] Steinberg MH, McCarthy WF, Castro O, Ballas SK (2010). The risks and benefits of long-term use of hydroxyurea in sickle cell anemia: A 17.5 year follow-up. Am J Hematol.

[3] Hanft VN, Fruchtman SR, Pickens CV, Rosse WF (2000). Acquired DNA mutations associated with *in vivo* hydroxyurea exposure. Blood.

[4] Friedrisch JR, Prá D, Maluf SW, Bittar CM (2008). DNA damage in blood leukocytes of individuals with sickle cell disease treated with hydroxyurea. Mutat. Res./Genet. Toxicol. Environ. Mutagen.

[5] Juul T, Malolepszy A, Dybkaer K, Kidmose R (2010). The *in vivo* toxicity of hydroxyurea depends on its direct target catalase. J Biol Chem.

[6] Ziegler-Skilkakis K, Schwarz LR, Andrae U (1985). Microsome- and hepatocyte-mediated mutagenicity of hydroxyurea and related aliphatic hydroxamic acids in V79 Chinese hamster cells. Mutat. Res.

[7] Evans RM (1985). Guidelines for handling parenteral antineoplastics. JAMA.

[8] Maron DN, Ames BN (1983). Revised methods for the Salmonella mutagenicity test. Mutat. Res.

[9] Bernstein L, Kaldor J, McCann J, Pike MC (1982). An empirical approach to the statistical analysis of mutagenesis data from the Salmonella test. Mutat. Res.

[10] Santos FV, Colus IMS, Silva MA, Vilegas W (2006). Assessment of DNA damage induced by extracts and fractions of Strychnos pseudoquina, a Brazilian medicinal plant with antiulcerogenic activity. Food Chem. Toxicol.

[11] Hayashi M, Morita T, Kodama Y, Sofundi T (1990). The micronucleus assay with mouse peripheral blood reticulocytes using acridine orange-coated slides. Mutat. Res.

[12] Bruce WR, Heddle JA (1979). The mutagenic activity of 61 agents as determined by the micronucleus, Salmonella and sperm abnormality assays. Can. J. Genet. Cytol.

[13] Lee M, Kwon J, Chung MK (2003). Enhanced prediction of potential rodent carcinogenicity by utilizing comet assay and apoptotic assay in combination. Mutat. Res.

